# Effects of cytokine-induced killer cell treatment combined with FOLFOX4 on the recurrence and survival rates for gastric cancer following surgery

**DOI:** 10.3892/etm.2013.1247

**Published:** 2013-08-05

**Authors:** HONGXIANG LIU, JUNMIN SONG, ZHEN YANG, XIEFU ZHANG

**Affiliations:** 1 Departments of Gastrointestinal Surgery, The First Affiliated Hospital of Zhengzhou University, Zhengzhou, Henan 450012, P.R. China; 2 Anorectal Surgery, The First Affiliated Hospital of Zhengzhou University, Zhengzhou, Henan 450012, P.R. China

**Keywords:** CIK cells, FOLFOX4, gastric cancer, recurrence rate, survival rate

## Abstract

The aim of this study was to investigate the effects of cytokine-induced killer (CIK) cell treatment combined with FOLFOX4 on the recurrence and survival rates of patients suffering from gastric cancer following surgery. A total of 98 patients with gastric cancer, who were surgically treated from June 2010 to June 2012, were divided into two groups: 47 patients, who underwent FOLFOX4 treatment alone, served as the control group, while the remaining 51 patients received FOLFOX4 in combination with CIK cell immunotherapy and served as the observation group. The immune functions, recurrence and survival rates were estimated and compared between the two groups. No significant differences were observed between the immune functions of the patients prior to treatment compared with the functions following treatment (P>0.05). However, the immune functions of the patients were improved following FOLFOX4 treatment in combination with CIK cell immunotherapy compared with the functions of the patients who received FOLFOX4 treatment alone (P<0.05). The gastric cancer recurrence rates of the patients in the observation group were significantly lower compared with those of the patients in the control group (5.9 versus 25.5, 17.6 versus 36.2 and 23.5 versus 48.9% after 1, 2 and 3 years, respectively; P<0.05). In addition, the survival rates of the patients with gastric cancer in the observation group were significantly enhanced compared with those of the control group, as assessed by log-rank test analysis (98.0 versus 93.6, 92.2 versus 78.7 and 72.5 versus 59.6% after 1, 2 and 3 years, respectively; P<0.05). It may be concluded that FOLFOX4 combined with CIK cell treatment has significant benefits for patients suffering from gastric cancer, compared with FOLFOX4 treatment alone.

## Introduction

Gastric cancer is one of the most common causes of mortality due to cancer in China (1). Surgery is the standard treatment procedure for localized and resectable gastric cancer. However, the survival rate of patients with advanced gastric cancer after surgery remains low. Decreasing the recurrence rates and extending the life span of patients suffering from gastric cancer has been the focus of a number of clinical investigations, with one study showing that chemotherapy may improve the survival rates of patients with gastric cancer postoperatively; however, the effectiveness was limited (2). Therefore, considerable efforts are required to improve the current therapeutic modalities and to explore novel therapies. In recent years, adoptive immunotherapy has been generally used in clinical practice. A number of adoptive immuno-therapies with killer cells have been developed, including tumor infiltrating lymphocyte (TIL), dendritic cell (DC) and cytokine-induced killer (CIK) cell therapies. At present, CIK cells are a novel type of antitumor effector cell that have been demonstrated to proliferate rapidly *in vitro*, with a stronger antitumor activity and a broader target tumor spectrum than the alternative antitumor effector cells that have been investigated (3,4). Moreover, CIK cells are able to regulate and enhance immune function (5).

In the present study, the potential benefits of the combination of autologous CIK cells with FOLFOX4 were investigated in patients suffering from gastric cancer and were compared with the effects of chemotherapy alone.

## Subjects and methods

### Clinical data

A total of 98 patients with gastric cancer who were treated surgically in The First Affiliated Hospital of Zhengzhou University (Zhengzhou, China) from June 2010 to June 2012 were enrolled in this study. The patients received a gastroscopy and a barium meal examination prior to the surgery. The 98 patients with gastric cancer were divided into two groups following the surgery: The control group (47 patients) were treated with FOLFOX4 alone, while the observation group (51 patients) were treated with FOLFOX4 in combination with CIK cell immunotherapy. The characteristics of the patients, such as gender, age, pathological grade, tumor site, histological type, invasion depth, lymph node metastasis and tumor stage, were collected and evaluated. The control group comprised 31 males and 16 females, aged from 35 to 76 years with a median age of 55.2±12.7 years. The cancer characteristics of the patients in the control group were as follows: pathological grade, 12 prophase and 35 aggressive phase; tumor site, 9 gastric fundus cardia, 20 gastric corpus and 18 gastric antrum; histological type, 21 moderately well-differentiated adenocarcinoma and 26 poorly differentiated adenocarcinoma; invasion depth, 24 with serosa infiltration and 23 without serosa infiltration; lymph node metastasis, 25 lymphaden transfer; and tumor stage, 6 stage I, 19 stage II, 18 stage III and 4 stage IV. The observation group comprised 34 males and 17 females, aged from 33 to 78 years with a median age of 56.1±11.9 years. The cancer characteristics of the patients in the observation group were as follows: pathological grade, 14 prophase and 37 aggressive phase; tumor site, 8 gastric fundus cardia, 23 gastric corpus and 20 gastric antrum; histological type, 21 moderately well-differentiated adenocarcinoma and 30 poorly differentiated adenocarcinoma; invasion depth, 23 with serosa infiltration and 28 without serosa infiltration; lymph node metastasis, 30 lymphaden transfer; tumor stage, 8 stage I, 20 stage II, 16 stage III and 7 stage IV. No statistical differences were identified between the two groups with regard to the gender and age of the patients, or the pathological grade, tumor site, histological type, invasion depth, lymph node metastasis or tumor stage (P>0.05). The study was conducted in accordance with the Declaration of Helsinki and with approval from the Ethics Committee of the First Affiliated Hospital of Zhengzhou University. Written informed consent was obtained from all participants.

### Treatments

Patients in the control group underwent two cycles of chemotherapy with the protocol of FOLFOX4 [oxaliplatin (L-OHP) 85 mg/m^2^, 3 h on day 1; calcium folinate (CF) 200 mg/m^2^, 2 h on days 1 and 2; and 5-fluorouracil (5-Fu) 400 mg/m^2^, 22 h on days 1 and 2]. One cycle comprised 14 days. All the patients in the two groups were treated with a 5-HT receptor blocker and vitamin B_6_ prior to therapy. Appropriate treatments were administered if the numbers of peripheral blood leucocytes or platelets, or the hemoglobin levels of the patients appeared to descend.

Patients in the observation group received CIK cell immunotherapy following two cycles of chemotherapy. All the patients underwent a routine blood examination after blood collection. A total of 50 ml blood was drawn from each patient using sodium citrate as an anticoagulant. Peripheral blood mononuclear cells (PBMCs) were isolated using hydroxypropylmethyl cellulose and were subsequently cultured in Medium I containing RPMI-1640 in the presence of human interferon-γ (IFN-γ, 1.0×10^6^ U/l), recombinant human interleukin-1α (IL-1α, 100 U/ml) and human IL-2 (5.0×10^5^ U/l). A monoclonal antibody (MAb) against CD3 (50 ng/ml) was added following 24 h of culture. The supernatant was aspirated and the cells were cultured in Medium II in the absence of INF-γ following a further 48 h culture. The cells were then cultured for 15 days and the medium was changed every 2 days. CIK cells (1.0×10^9^) were transfused into the patients for 1 h every second day. The curative efficacy was evaluated subsequent to each treatment. The cells were identified and sorted by flow cytometry using a fluorescence-activated cell sorter (FACS; Beckman-Coulter, Miami, FL, USA) on days 1, 5, 10, 15, and 20. In addition, the whole blood cells of the patients were separated and sorted by FACS prior to and following treatment.

### Evaluation

The patients were followed up from June 2010 to June 2012, and were assessed every 3 months for the first year, then every 6 months for the second and third years. The immune functions [CD3, CD4, CD8, CD4/CD8 and natural killer (NK) cells], cumulative recurrence rates, cumulative survival rates and the survival times of the two groups were analyzed prior to and following treatment.

### Statistical analysis

Statistical analysis was performed using SPSS version 3.0 statistical software (SPSS, Inc., Chicago, IL, USA). All values are expressed as the mean ± standard deviation. Comparisons were made using the Student’s t-test or the χ^2^ test. The cumulative recurrence and survival curves were estimated using the Kaplan-Meier method and the differences in the distributions were compared with the log-rank test. P<0.05 was considered to indicate a statistically significant difference.

## Results

### Analysis of CIK cell phenotype

**Figure 1.**
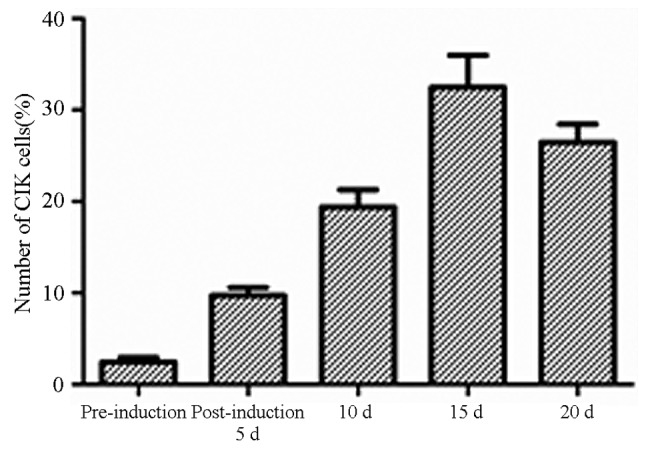
Change in cytokine-induced killer (CIK) cell percentage at different stimulation time-points.

CD3^+^ CD56^+^ CIK cells were induced from the PBMCs of the patients following stimulation by INF-γ, IL-1α, IL-2 and anti-CD3 MAb. The number of CIK cells gradually increased the during cell culture, peaking on day 15 and then decreasing slightly with further culture (Fig 1).

### Comparison of the immune function of the patients prior to and following treatment

No statistical differences were observed in the levels of CD3^+^, CD4^+^, CD8^+^, CD4^+^/CD8^+^ and NK cells between the two groups prior to treatment (P>0.05). Moreover, there were no statistically significant differences between the levels of CD3^+^, CD4^+^, CD8^+^, CD4^+^/CD8^+^ and NK cells in the control group prior to and following treatment (P>0.05). However, the levels of CD3^+^, CD4^+^, CD8^+^, CD4^+^/CD8^+^ and NK cells in the observation group following treatment were markedly higher than those prior to treatment and were also significantly enhanced compared with the levels in the control group following treatment, and the level of CD8+ in the observation group following treatment was markedly lower than those prior to treatment (P<0.05; Table I).

### Comparison of the recurrence rates of the two groups

**Figure 2.**
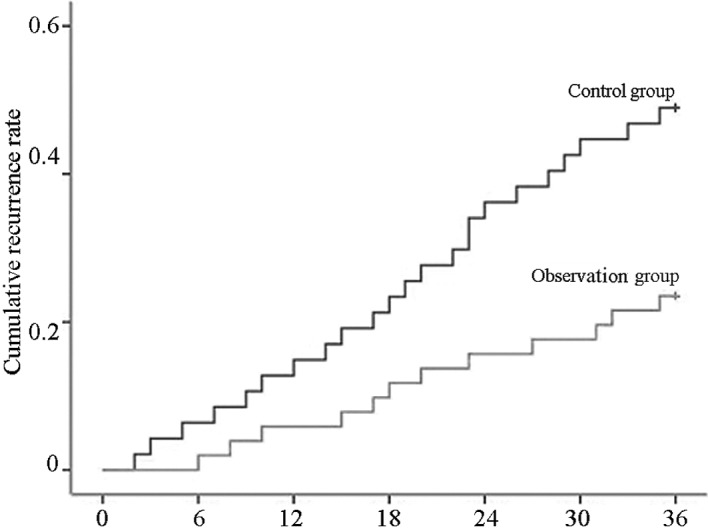
Comparison of the recurrence rates of the control and observation groups at 1, 2 and 3 years.

There were 23 postoperative recurrences in the control group, and 12 in the observation group. The log-rank test demonstrated that the gastric cancer recurrence rates of the patients in the observation group were significantly lower than those of the patients in the control group (5.9 versus 25.5, 17.6 versus 36.2 and 23.5 versus 48.9% for the observation and control groups following 1, 2 and 3 years, respectively; P<0.05; Fig. 2).

### Comparison of the survival rates of the two groups

**Figure 3.**
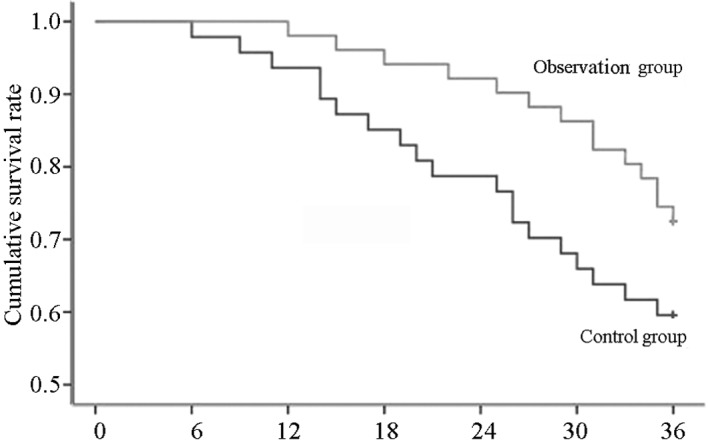
Comparison of the survival rates of the control and observation groups at 1, 2 and 3 years.

In the observation group, there were 19 fatalities and the average survival time was 21 months, while in the control group, there were 11 fatalities and the average survival time was 29 months. The survival rates of the patients with gastric cancer in the observation group were demonstrated to be significantly enhanced in comparison with those in the control group following analysis with a log-rank test (98.0 versus 93.6, 92.2 versus 78.7 and 72.5 versus 59.6% after 1, 2 and 3 years, respectively; P<0.05; Fig. 3).

## Discussion

Despite the standardization of surgery and multimodal therapies, the postoperative survival of patients with advanced gastric cancer remains poor. Previous studies have shown that the recurrence rates are 2–14% in early gastric cancer, more than 50% in advanced gastric carcinoma and ∼30% in the patients with a 5-year survival (6,7). It has been demonstrated that adjuvant chemotherapy for gastric cancer following curative resection may improve the disease-free and overall survival times of patients with gastric cancer. However, numerous cycles of chemotherapy may lead to a reduction in the immune functions of the patients with gastric cancer, with decreased ratios of CD4^+^ and CD4^+^/CD8^+^ cells, reduced NK cell activities and an increased proportion of CD8^+^ cells (8). These observations are consistent with those of the present study. Therefore, novel therapies that significantly improve the immune functions of patients with cancer are required.

In recent years, immunotherapy has become the fourth most important treatment modality for malignant tumors (9). It has been shown that cellular immunotherapy is able to promote host anticancer immunity, thus prolonging the survival time of patients with gastric cancer. The treatment of gastric cancer with autologous CIK cells is one such promising cellular immunotherapy (9–12). It has been demonstrated that CIK cells proliferate abundantly *in vitro* and kill tumor cells directly. Moreover, CIK cells are able to regulate and increase the host cellular immune function *in vivo* (13–16). The results of the present study suggest that CIK cell immunotherapy is able to significantly improve the immune functions of patients with cancer, showing that the proportions of the CD3^+^ and CD4^+^ T-cell subgroups were significantly increased, the activities of the NK cells were enhanced, the proportion of CD8^+^ cells was markedly decreased and the ratio of CD4^+^/CD8^+^ cells was normal following CIK cell immunotherapy.

The current study also investigated the effects of CIK cell treatment combined with FOLFOX4 on the recurrence and survival rates of the patients with gastric cancer. The results showed that in years 1, 2 and 3, for the control group, the cumulative recurrence rates were 25.5, 36.2 and 48.9%, respectively and the survival rates were 93.6, 78.7 and 59.6% respectively, while for the observation group, the recurrence rates were 5.9, 17.6 and 23.5%, respectively and the survival rates were 98.0, 92.2 and 72.5%, respectively. It was observed that the recurrence rates of the observation group were lower compared with those of the control group, whereas the survival rates of the observation group were higher than those of the control group. The results suggested that CIK cell immunotherapy combined with chemotherapy may have a synergistic effect.

In conclusion, adjuvant immunotherapy with CIK cells significantly reduced the recurrence rate and prolonged the survival time of patients with gastric cancer. Furthermore, the treatment with CIK cells combined with FOLFOX4 demonstrated notable efficacy in improving the immune function of the patients.

## Figures and Tables

**Table I.** Phenotypic analysis of cytokine-induced killer cells of the control and observation groups pretherapy and 10 days post-therapy.

Group	n	CD3^+^ (%)	CD4^+^ (%)	CD8^+^ (%)	CD4^+^/CD8^+^ (%)	NK cells (%)
Control						
Pretherapy	47	57.04±10.23	28.99±8.14	25.79±6.41	0.96±0.31	17.89±5.89
Post-therapy	47	55.39±8.90	18.16±8.54	26.39±7.01	1.02±0.26	18.14±6.01
Observation						
Pretherapy	51	57.21±9.44	29.54±7.82	26.32±6.48	1.06±0.25	18.23±6.12
Post-therapy	51	66.75±9.81^ab^	38.12±8.02^ab^	19.62±5.92^ab^	1.37±0.29^ab^	26.11±6.48^ab^

a P<0.05 vs. pretherapy value;

b P<0.05 vs. the control group.

NK, natural killer.
